# Effects of rumen-protected creatine pyruvate on blood biochemical parameters and rumen fluid characteristics in transported beef cattle

**DOI:** 10.1186/s12917-021-03134-y

**Published:** 2022-01-15

**Authors:** Kang Mao, Guwei Lu, Yanjiao Li, Yitian Zang, Xianghui Zhao, Qinghua Qiu, Mingren Qu, Kehui Ouyang

**Affiliations:** 1 Jiangxi Province Key Laboratory of Animal Nutrition, College of Animal Science and Technology, Jiangxi Agricultural University, Nanchang, China; 2 Jiangxi Province Key Laboratory of Animal Nutrition/Animal Nutrition and Feed Safety Innovation Team, College of Animal Science and Technology, Jiangxi Agricultural University, Nanchang, China; 3 College of Animal Science and Technology, Jiangxi Agricultural University, Nanchang, China

**Keywords:** Antioxidant activity, Beef cattle, Creatine pyruvate, Rumen fermentation transport stress

## Abstract

**Background:**

The fasting and stress associated with road transportation contributes to a lack of energy and a decline in the immune system of beef cattle. Therefore, it is essential for beef cattle to enhance energy reserves before transportation. Creatine pyruvate (CrPyr) is a new multifunctional nutrient that can provide both pyruvate and creatine, which are two intermediate products of energy metabolism. To investigate the effects of transport and rumen-protected (RP)-CrPyr on the blood biochemical parameters and rumen fluid characteristics of beef cattle, twenty male Simmental crossbred cattle (659 ± 16 kg) aged 18 months were randomly allocated to four groups (*n* = 5) using a 2 × 2 factorial arrangement with two RP-CrPyr supplemental levels (0 or 140 g/d) and two transport treatments (5 min or 12 h): T_CrPyr140, T_CrPyr0, NT_CrPyr140, and NT_CrPyr0. After feeding for 30 days, three cattle per treatment were slaughtered.

**Results:**

Compared with nontransport, transport decreased the total antioxidant capacity, catalase activity, contents of IgA, interferon γ, interleukin-1β (IL-1β), and IL-6 in serum, and the amounts of total volatile fatty acids (TVFA), acetate, and butyrate in rumen (*P* < 0.05); increased the serum lipopolysaccharide (LPS) level, contents of rumen LPS and ammonia nitrogen (*P* < 0.05). RP-CrPyr supplementation decreased the levels of cortisol and LPS in serum and the butyrate concentration in the rumen of beef cattle compared with those in the unsupplemented groups (*P* < 0.05). RP-CrPyr and transport interaction had a significant effect on the contents of serum tumour necrosis factor-α, IL-6, LPS, ruminal pH, acetate content, and acetate/propionate (*P* < 0.05). In terms of ruminal bacterial composition, group T_CrPyr0 increased the *Prevotella* genus abundance compared with group NT_CrPyr0 (*P* < 0.05), while group T_CrPyr140 increased Firmicutes phylum abundance and decreased Bacteroidetes phylum and genus *Prevotella* abundance compared with group T_CrPyr0 (*P* < 0.05). Moreover, Bacteroidetes was positively correlated with serum LPS.

**Conclusions:**

These results indicated that dietary supplementation with RP-CrPyr might be beneficial to alleviate transport stress by decreasing serum cortisol and LPS levels and promoting the restoration of the rumen natural flora.

## Background

In China, allochthonous fattening is the dominant beef cattle production model. The north reproduction and south raise have become inherent patterns. The fasting and stress associated with long-distance transportation contributes to a lack of energy and a decline in the immune system of beef cattle. Subsequently, transport stress syndrome of beef cattle (TSSBC) with significant morbidity and high mortality rates frequently occurs following long-distance transportation and leads to enormous economic losses [1, 2]. Ruminal microecological balance is essential for adequate nutrient supply and immunity improvement. Microbial fermentation of feedstuff in the rumen produces volatile fatty acids (VFAs) and microbial protein to provide the bulk of the energy and protein required by the host animal [3]. However, several studies show that transport stress affects ruminal microbiota abundance and diversity [4, 5], which might further influence host feed digestion and metabolism. Moreover, animals exposed to stress emit gastrointestinal pathogens that can increase the secretion of virulence factors [6]. Thus, it is essential for beef cattle to enhance energy reserves before transportation and maintain the ruminal microecological balance and function during transportation to improve ruminant health.

Creatine pyruvate (CrPyr), with a molecular formula of C_7_H_13_N_3_O_5_ and a molecular weight of 219.20, is a new multifunctional nutrient that contains pyruvate and creatine at a ratio of 40:60 and can provide both pyruvate and creatine [7]. For humans and nonruminants, several studies have shown that CrPyr can enhance body energy metabolism. Jäger et al. [8] reported that CrPyr could promote aerobic metabolism in athletes to enhance endurance performance. Chen [9] found that CrPyr supplementation increased the creatine kinase activity and phosphocreatine concentration in the muscle of broilers and increased glycogen reserve in muscle by reducing the decomposition of glycogen by decreasing phosphorylase-b activity. Recently, it was reported that *in ovo* feeding of CrPyr enhanced energy reserves in the liver and muscle of broilers on the day of hatching, increased glycolytic enzyme activity and promoted glycolysis in the chest [10]. In addition, our previous study showed that nonbypassed CrPyr could relieve the heat stress of beef cattle by improving antioxidant activity and rumen microbial protein synthesis [11]. Given these properties of CrPyr, we investigated the influences of RP-CrPyr (rumen bypass ratio is 82.8%) on serum hormone levels, antioxidant activity, immunity, rumen fermentation, and the ruminal microorganism community of cattle with transport treatment. We hypothesized that the present work will lay a foundation for the further study of RP-CrPyr supplementation in the transport stress of cattle. Therefore, this study was designed to test this hypothesis.

## Results

### Serum hormones and antioxidant parameters

The results of serum hormones and antioxidant parameters are presented in Table 1. Compared with the nontransport treatment, transport significantly decreased the serum T-AOC (1.98 vs. 2.03 mmol/L; *P* < 0.01) and CAT activity (28.41 vs. 40.53 U/mL; *P* < 0.05) in beef cattle. Supplementation with RP-CrPyr significantly decreased the concentrations of serum cortisol (62.63 vs. 80.58 ng/mL; *P* < 0.05) and tended to decrease the concentrations of serum T3 (1.10 vs. 1.18 ng/mL; *P* = 0.081) and MDA (4.90 vs. 6.99 nmol/mL; *P* = 0.088) compared with cattle given 0 g/day RP-CrPyr treatment. There was no interaction on serum antioxidant parameters.

**Table 1:** Effects of transport and rumen-protected creatine pyruvate on the serum hormones and antioxidant parameters in the beef cattle

Item	Treatment^a^				SEM	*P*-value		
	Transport		Nontransport					
	RP-CrPyr_140_	RP-CrPyr_0_	RP-CrPyr_140_	RP-CrPyr_0_		Transport	RP-CrPyr	Transport × RP-CrPyr^b^
T3 (ng/mL)	1.12	1.22	1.07	1.14	0.031	0.151	0.081	0.732
T4 (ng/mL)	74.65	72.16	68.39	76.90	5.682	0.815	0.365	0.117
Cortisol (ng/mL)	62.99	84.66	62.27	76.49	12.620	0.430	0.010	0.505
MDA (nmol/mL)	5.61	7.92	4.19	6.07	0.539	0.167	0.088	0.853
SOD (U/mL)	88.77	80.15	85.60	84.41	2.165	0.908	0.317	0.442
T-AOC (mmol/L)	2.00	1.98	2.04	2.02	0.005	0.002	0.117	0.882
GSH-Px (U/mL)	109.65	125.14	133.14	136.24	6.864	0.174	0.607	0.445
CAT (U/mL)	27.15	24.50	41.36	39.68	3.253	0.010	0.634	0.914

^a^ A 2 × 2 factorial arrangement with 2 supplemental levels of rumen-protected creatine pyruvate (RP-CrPyr) in basal diets (0 or 140 g/d for each cattle) and 2 transport treatment (transport 0 or 12 h before slaughter)

^b^ Transport × RP-CrPyr, the interaction between Transport and RP-CrPyr

*SEM* Stand error of mean, *T3* Triiodothyronine, *T4* Thyroxine, *MDA* Malondialdehyde, *SOD* Superoxide dismutase, *T-AOC* Total antioxidant capacity, *GSH-Px* Glutathione per-oxidase, *CAT* Catalase

### Immunoglobulin, inflammatory factor, lipopolysaccharide, and extracellular DNA levels

As shown in Table 2, compared with the nontransport treatment, the contents of serum IgA (0.35 vs. 0.45 g/L), IFN-γ (32.69 vs. 44.54 pg/mL), IL-1β (20.87 vs. 29.86 pg/mL), and IL-6 (100.37 vs. 138.97 pg/mL) were significantly decreased (*P* < 0.05), and the levels of ruminal LPS (2.30 vs. 0.76 EU/mL; *P* < 0.001) and serum LPS (22.94 vs. 10.98 EU/mL; *P* < 0.01) were significantly increased in beef cattle in the transport treatment. However, transport treatment did not significantly affect the contents of IgG, IgM, and TNF-α in serum. Moreover, there was no significant difference in the levels of immunoglobulin and inflammatory factors between the RP-CrPyr-supplemented and unsupplemented groups (*P* > 0.05). However, dietary RP-CrPyr supplementation decreased the serum LPS level (13.85 vs. 20.07 EU/mL; *P* < 0.05) of beef cattle than those in the unsupplemented groups. Transport or RP-CrPyr did not affect the contents of eDNA in serum or rumen fluid (*P* > 0.05). RP-CrPyr and transport interaction had a significant effect on serum TNF-α and IL-6 (*P* < 0.05).

**Table 2:** Effects of transport and rumen-protected creatine pyruvate on the contents of immunoglobulin and inflammatory factor in serum, lipopolysaccharide and extracellular DNA in serum and rumen fluid of beef cattle

Item	Treatment^1^				SEM	*P*-value		
	Transport		Nontransport					
	RP-CrPyr_140_	RP-CrPyr_0_	RP-CrPyr_140_	RP-CrPyr_0_		Transport	RP-CrPyr	Transport × RP-CrPyr^2^
*Serum*								
IgG (g/L)	9.44	8.75	9.93	9.80	0.607	0.539	0.744	0.820
IgM (g/L)	2.44	2.36	2.59	2.47	0.167	0.689	0.776	0.960
IgA (g/L)	0.34	0.36	0.44	0.47	0.021	0.028	0.543	0.983
TNF-α (pg/mL)	59.24	47.91	46.75	57.89	2.594	0.791	0.984	0.040
IFN-γ (pg/mL)	31.92	33.45	41.83	47.25	1.573	0.005	0.301	0.554
IL-1β (pg/mL)	22.87	18.86	28.83	30.89	1.643	0.026	0.773	0.383
IL-6 (pg/mL)	87.00^c^	113.72^bc^	149.19^a^	128.75^ab^	4.301	0.002	0.725	0.025
LPS (EU/mL)	16.71^b^	29.18^a^	11.00^b^	10.96^b^	2.463	0.001	0.032	0.031
eDNA (ng/μL)	2.36	2.39	2.11	2.17	0.146	0.459	0.882	0.975
*Rumen*								
LPS (EU/mL)	2.19	2.41	0.64	0.89	0.074	< 0.001	0.143	0.929
eDNA (ng/μL)	19.95	20.94	26.12	17.00	1.503	0.720	0.213	0.131

^a,b,c^ Means within a row with no common superscript differ significantly (*P* < 0.05)

^1^ A 2 × 2 factorial arrangement with 2 supplemental levels of rumen-protected creatine pyruvate (RP-CrPyr) in basal diets (0 or 140 g/d for each cattle) and 2 transport treatment (transport 0 or 12 h before slaughter)

^2^ Transport × RP-CrPyr, the interaction between Transport and RP-CrPyr

*SEM* Stand error of mean, *TNF-α* Tumor necrosis factor-α, *IFN-γ* Interferon γ, *IL-1β* Interleukin-1β, *IL-6* Interleukin-6, *LPS* Lipopolysaccharide, *eDNA* Extracellular DNA

### Rumen fermentation characteristics

The results of rumen fermentation characteristics are presented in Table 3. Compared with the nontransport treatment, transport tended to increase the rumen fluid pH (6.80 vs. 6.51; *P* = 0.087) and decrease ruminal propionate content (11.25 vs. 12.61 mmol/L; *P* = 0.070); significantly increased the ruminal NH_3_-N content (18.78 vs. 8.22 mg/dL; *P* < 0.001), decreased the concentrations of ruminal TVFA (76.73 vs. 86.49 mmol/L; *P* < 0.01), acetate (58.36 vs. 64.26 mmol/L; *P* < 0.05) and butyrate (7.11 vs. 9.92 mmol/L; *P* < 0.001). Supplementation with RP-CrPyr tended to decrease the concentration of ruminal VFAs (79.12 vs. 84.10 mmol/L; *P* = 0.050) and significantly decrease the ruminal butyrate content (7.44 vs. 9.59 mmol/L; *P* < 0.001). Transport or RP-CrPyr did not affect the content of ruminal MCP (*P* > 0.05). Except for rumen fluid pH, ruminal acetate, acetate/propionate, and serum LPS, there was no significant RP-CrPyr and transport interaction effect on the other characteristics.

**Table 3:** Effects of transport and rumen-protected creatine pyruvate on the rumen fermentation characteristics of beef cattle

Item	Treatment^1^				SEM	*P*-value		
	Transport		Nontransport					
	RP-CrPyr_140_	RP-CrPyr_0_	RP-CrPyr_140_	RP-CrPyr_0_		Transport	RP-CrPyr	Transport × RP-CrPyr^2^
pH	7.02	6.58	6.37	6.65	0.076	0.087	0.619	0.046
MCP (mg/mL)	0.22	0.31	0.23	0.19	0.021	0.188	0.639	0.092
NH_3_-N (mg/dL)	16.64	20.93	8.18	8.26	0.672	< 0.001	0.143	0.155
TVFA (mmol/L)	72.18	81.28	86.06	86.92	1.997	0.001	0.050	0.060
Acetate (mmol/L)	54.75^b^	61.98^a^	66.21^a^	62.32^a^	5.374	0.026	0.461	0.033
Propionate (mmol/L)	11.35	11.15	11.67	13.56	1.383	0.070	0.231	0.528
Butyrate (mmol/L)	6.08	8.15	8.81	11.04	1.910	< 0.001	< 0.001	0.819
Acetate/Propionate	4.89	5.61	4.60	5.68	0.201	0.755	0.618	0.029

^a,b,c^ Means within a row with no common superscript differ significantly (*P* < 0.05)

^1^ A 2 × 2 factorial arrangement with 2 supplemental levels of rumen-protected creatine pyruvate (RP-CrPyr) in basal diets (0 or 140 g/d for each cattle) and 2 transport treatment (transport 0 or 12 h before slaughter)

^2^ Transport × RP-CrPyr, the interaction between Transport and RP-CrPyr

*SEM* Stand error of mean, *MCP* Microbial crude protein, *NH*_*3*_*-N* Ammonia nitrogen, *TVFA* Total volatile fatty acids

### Alpha-Diversity measures and OTU analysis

**Fig. 1:**
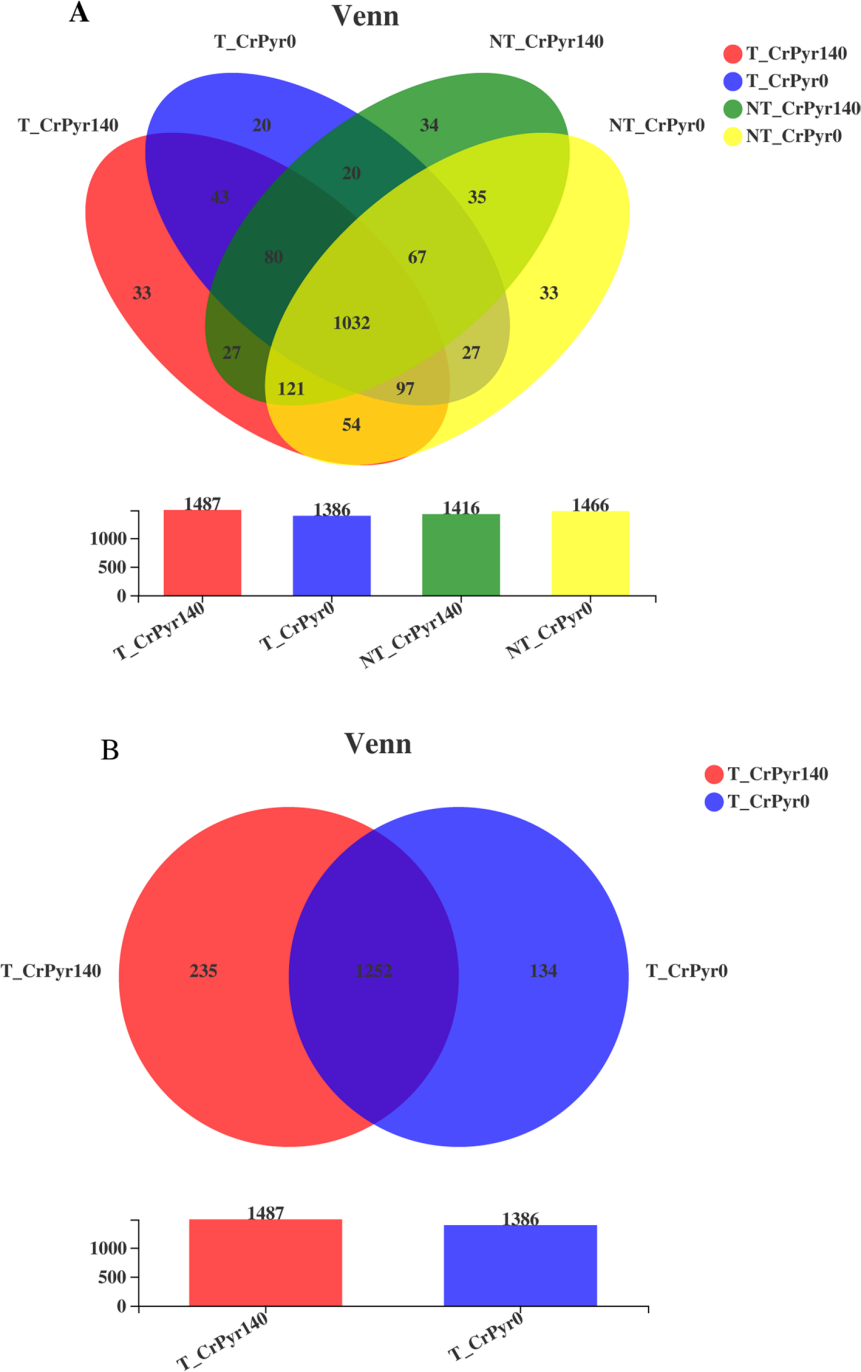
Operational taxonomic unit (OTU) Venn diagram among different groups. T_CrPyr140: Transport treatment + 140 g/d/cattle rumen-protected creatine pyruvate; T_CrPyr0: Transport treatment + 0 g/d/cattle rumen-protected creatine pyruvate. NT_CrPyr140: Nontransport treatment + 140 g/d/cattle rumen-protected creatine pyruvate; NT_CrPyr0: Nontransport treatment + 140 g/d/cattle rumen-protected creatine pyruvate

The alpha diversity index is mainly used to evaluate the richness and uniformity of microorganisms in samples. As shown in Table 4, at the same sequencing depth, the alpha values of the four groups were not significantly different in Ace, Chao1, Shannon, and Simpson (*P* > 0.05). Based on the principle of similarity greater than 97%, 1723 OTUs were obtained by the Venn diagram (Fig. 1A). Group T_CrPyr140 shared 1252 OTUs with group T_CrPyr0, and 235 and 134 OTUs were unique to group T_CrPyr140 and group T_CrPyr0, respectively (Fig. 1B).

**Table 4:** Effects of transport and rumen-protected creatine pyruvate on the Alpha diversity indices

Item	Treatment^a^				SEM	*P*-value		
	Transport		Nontransport					
	RP-CrPyr_140_	RP-CrPyr_0_	RP-CrPyr_140_	RP-CrPyr_0_		Transport	RP-CrPyr	Transport × RP-CrPyr^b^
Shannon	5.42	5.15	5.02	5.33	0.079	0.520	0.918	0.102
Simpson	0.01	0.02	0.02	0.01	0.002	0.805	0.599	0.223
Chao1	1274.40	1215.06	1155.33	1200.78	23.970	0.202	0.888	0.306
ACE	1269.03	1194.46	1140.30	1191.49	23.415	0.197	0.809	0.216

^a^ A 2 × 2 factorial arrangement with 2 supplemental levels of rumen-protected creatine pyruvate (RP-CrPyr)r in basal diets (0 or 140 g/d for each cattle) and 2 transport treatment (transport 0 or 12 h before slaughter)

^b^ Transport × RP-CrPyr, the interaction between Transport and RP-CrPyr

**Fig. 2:**
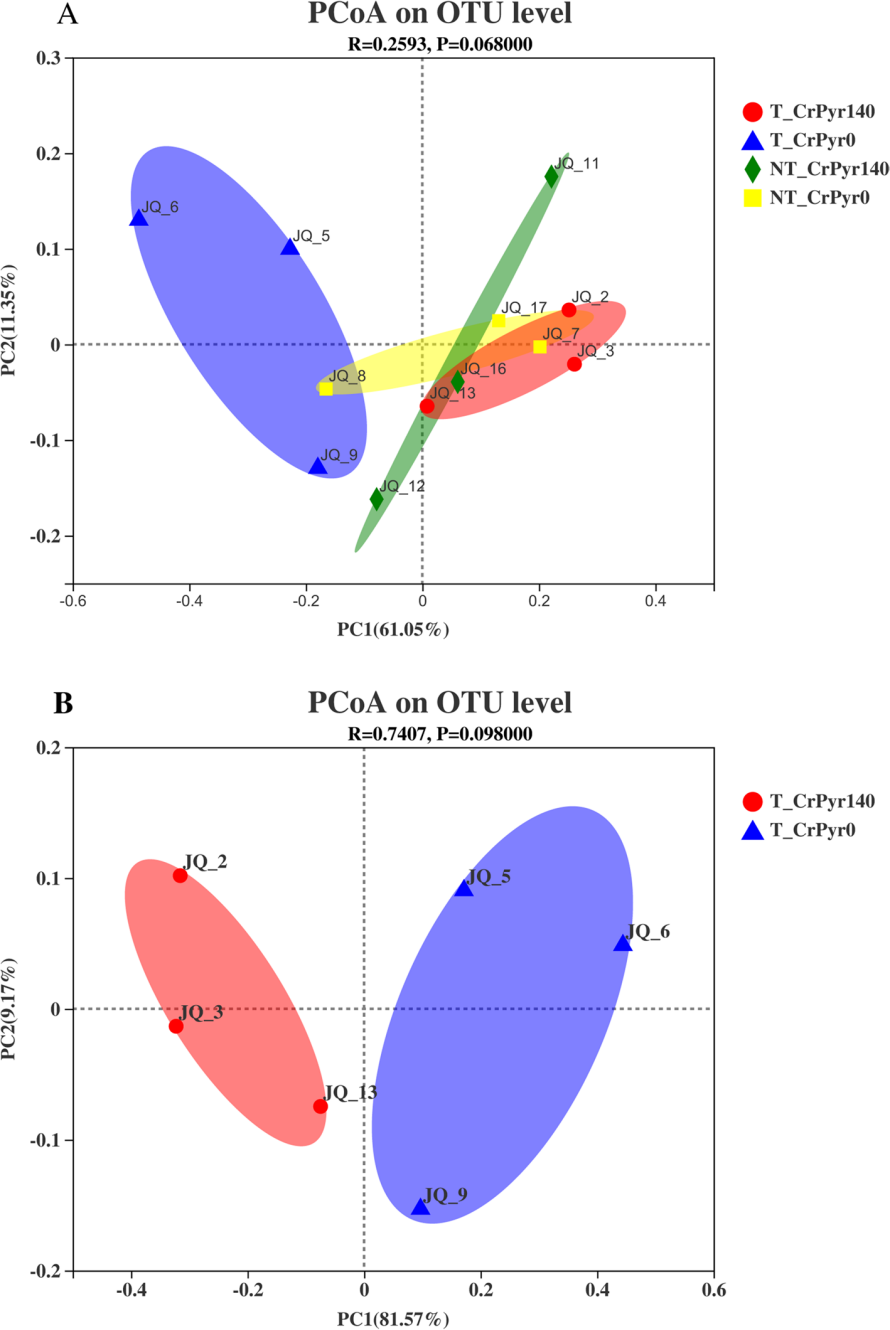
Principal coordinate analysis (PCoA) plot based on OTU abundance. T_CrPyr140: Transport treatment + 140 g/d/cattle rumen-protected creatine pyruvate; T_CrPyr0: Transport treatment + 0 g/d/cattle rumen-protected creatine pyruvate. NT_CrPyr140: Nontransport treatment + 140 g/d/cattle rumen-protected creatine pyruvate; NT_CrPyr0: Nontransport treatment + 140 g/d/cattle rumen-protected creatine pyruvate

To measure the extent of similarity between the microbial communities, beta diversity was calculated using a weighted normalized UniFrac, and PCoA was performed. As shown in Fig. 2, these microbial community profiles from the four groups were not significantly different (*P* = 0.068), but the distance between group T_CrPyr0 and group T_CrPyr140 was obviously separated, which indicated that with transport treatment, RP-CrPyr supplementation could affect the composition of the flora in rumen fluid compared with the RP-CrPy unsupplemented group.

### Bacterial composition

**Fig. 3:**
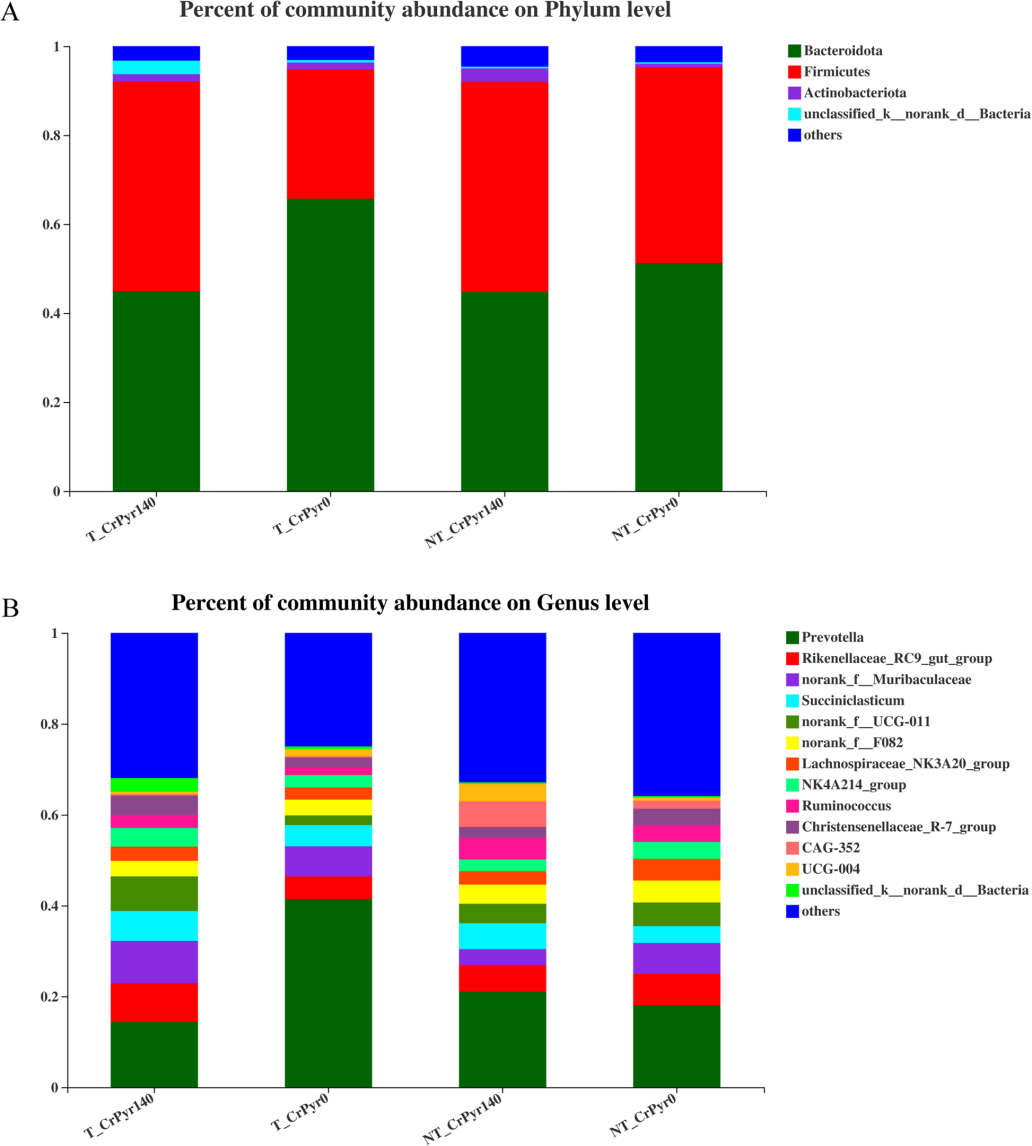
Bar plots showing the average relative abundance of bacterial phyla (%) in the rumen at the phylum level (**A**) and genus level (**B**). Data represent the abundance at greater than 0.03% of the community among these four groups. T_CrPyr140: Transport treatment + 140 g/d/cattle rumen-protected creatine pyruvate; T_CrPyr0: Transport treatment + 0 g/d/cattle rumen-protected creatine pyruvate. NT_CrPyr140: Nontransport treatment + 140 g/d/cattle rumen-protected creatine pyruvate; NT_CrPyr0: Nontransport treatment + 140 g/d/cattle rumen-protected creatine pyruvate

**Fig. 4:**
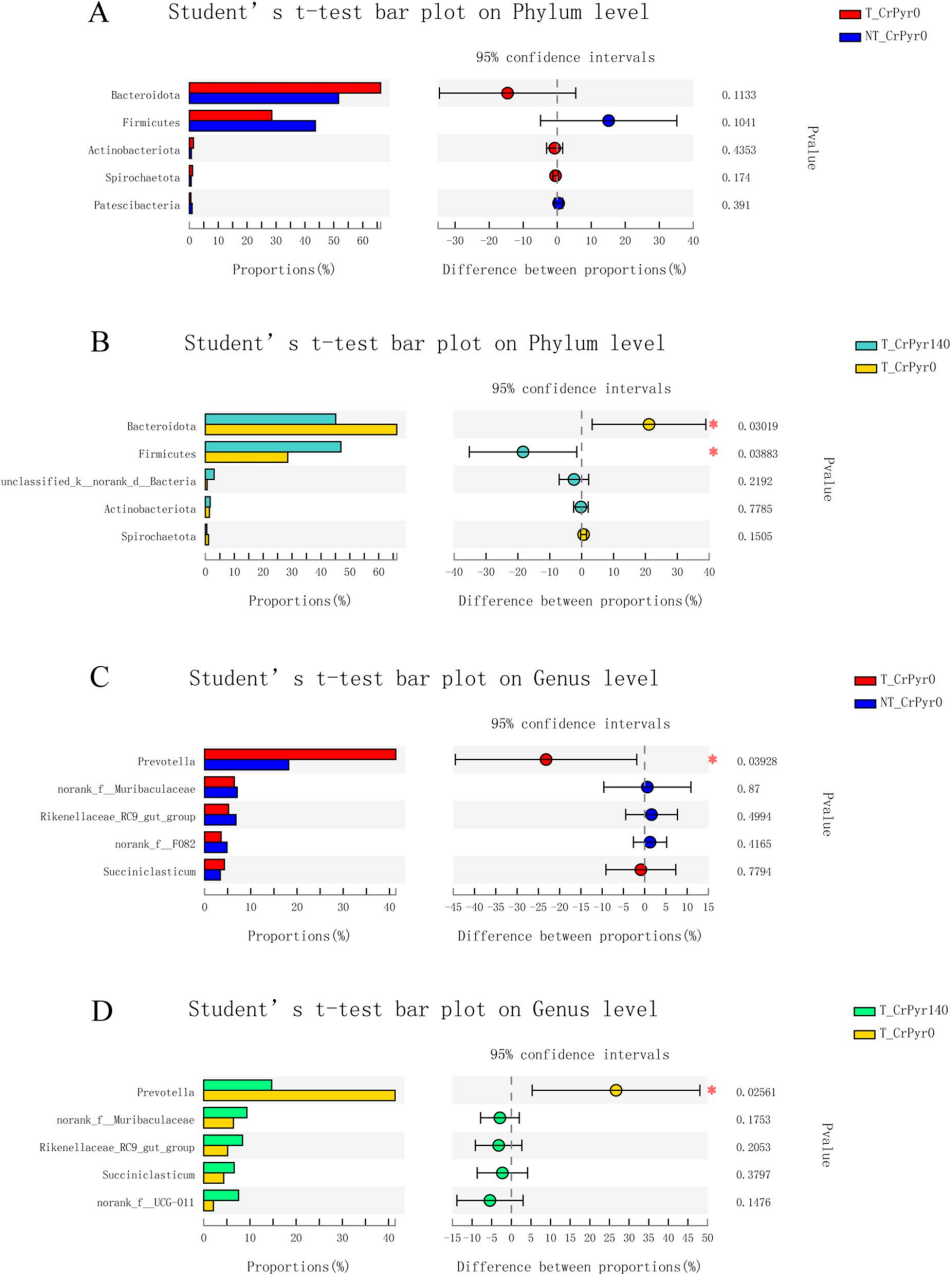
Analysis of species composition difference. At the phylum level, group T_CrPyr0 vs group NT_CrPyr0 (**A**) and group T_CrPyr140 vs group T_CrPyr0. At the genus level, compare between T_CrPyr0 and NT_CrPyr0 (**C**), T_CrPyr140 and T_CrPyr0 (**D**). T_CrPyr140: Transport treatment + 140 g/d/cattle rumen-protected creatine pyruvate; T_CrPyr0: Transport treatment + 0 g/d/cattle rumen-protected creatine pyruvate. NT_CrPyr140: Nontransport treatment + 140 g/d/cattle rumen-protected creatine pyruvate; NT_CrPyr0: Nontransport treatment + 140 g/d/cattle rumen-protected creatine pyruvate

Phylogenetic analysis identified nineteen phyla from the rumen fluid, four of which had a relative abundance > 0.3% of the community, and the most abundant phyla were Bacteroidetes, Firmicutes, Actinobacteria, and Unclassified_k_norank_d_Bacteria (Fig. 3A), and their relative abundance showed no significant difference among the four groups. With transport treatment, group T_CrPyr140 decreased Bacteroidetes abundance and increased Firmicutes abundance compared with group T_CrPyr0 (Fig. 4B, *P* < 0.05).

At the genus level, 247 genera were shown in the ruminal fluid, thirteen of which had a relative abundance greater than 0.3% of the community, including *Prevotella*, *Rikenellaceae_RC9_gut_group*, *norank_f_Muribaculaceae*, *Succiniclasticum*, *norank_f_UCG_-011*, *norank_f_F082*, *Lachnospiraceae_NK3A20_group*, *NK4A214_group*, *Ruminococcus*, *Christensenellaceae_R-7_group*, *CAG-352*, *UCG-004*, and *unclassified_k_norank_d_Bacteria* (Fig. 3B). Among them, group T_CrPyr0 increased the ruminal *Prevotella* abundance of beef cattle compared with group NT_CrPyr0 (Fig. 4C, *P* < 0.05), while group T_CrPyr140 decreased the *Prevotella* abundance compared with group T_CrPyr0 (Fig. 4D, *P* < 0.05).

### Correlation between rumen microbiota and physiological variables

**Fig. 5:**
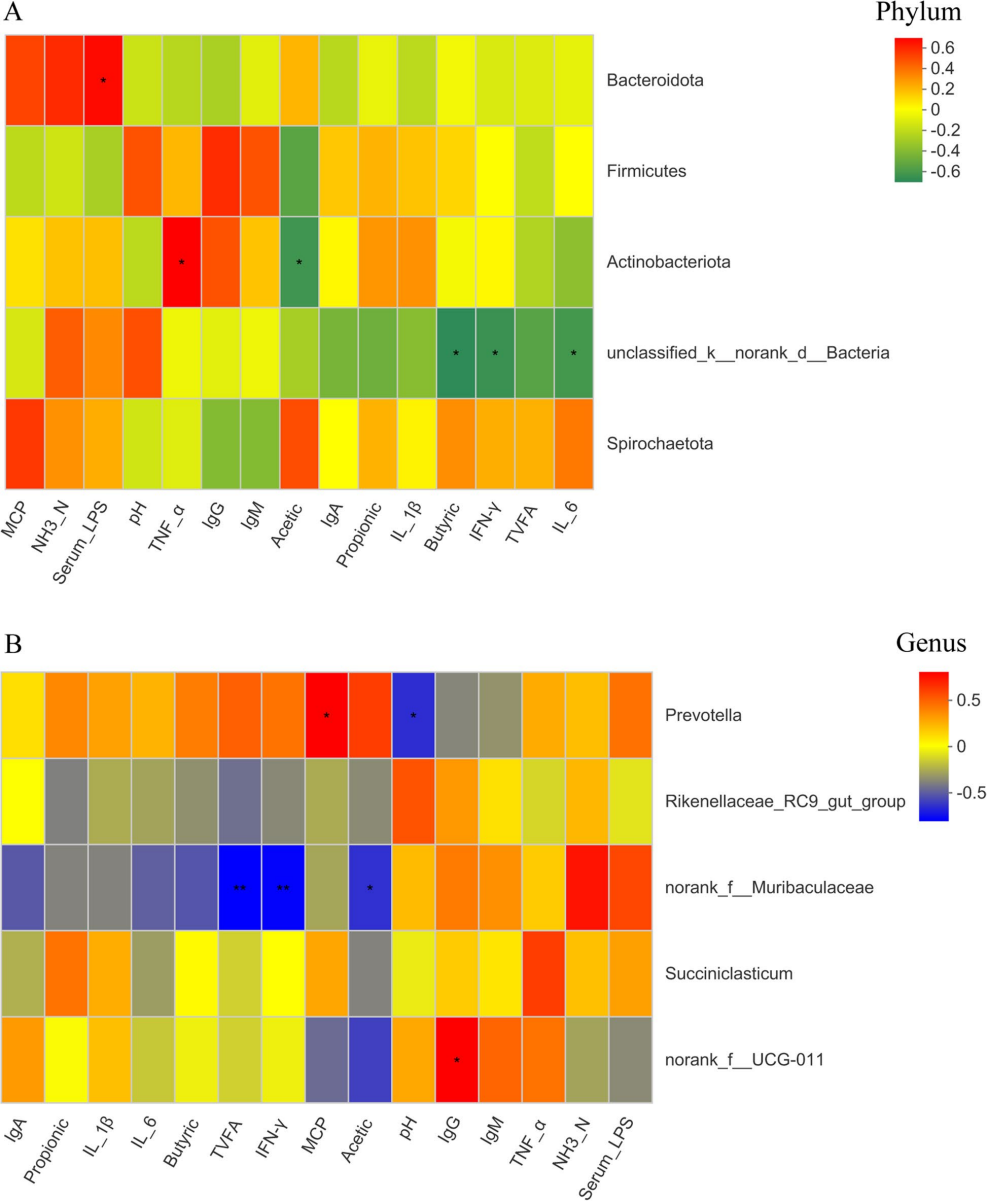
Spearmen’s correlation between the rumen bacterial communities and cattle physiological variables. The strength of the correlation between variables is represented by the size and color strength of the square (red: positive correlation; blue: negative correlation; white: no correlation)

The relationship between ruminal microbiota abundance (top 5 species) and physiological parameters was used to analyse the evaluated correlations. At the phylum level (Fig. 5A), serum LPS was positively correlated with Bacteroidetes (*P* < 0.05). The rumen fermentation characteristics of ruminal acetate were negatively correlated with Actinobacteriota (*P* < 0.05). For the immune system, serum TNF-α was positively correlated with Actinobacteriota. The abundance of unclassified_k_norank_d_bacteria was negatively correlated with serum IL-6. At the genus level (Fig. 5B), the abundance of *Prevotella* was negatively correlated with ruminal pH but positively correlated with ruminal MCP (*P* < 0.05). The rumen fermentation characteristics of acetate and TVFA were negatively correlated with *norank_f_Muribaculaceae* (*P* < 0.05). IgG was positively correlated with *norank_f_UCG-011* (*P* < 0.05).

## Discussion

During long-distance transportation, beef cattle encounter continuous and strong stimuli such as driving, loading and unloading, vibration, crowding, noise, temperature and humidity changes, water and food restriction, fatigue, etc. In response to transport stress, the hypothalamic–pituitary–adrenal (HPA) axis and/or sympathetic nervous system (SNS) were activated, which in turn regulated the corresponding hormones secreted by the endocrine glands. Of these, cortisol is often elevated in the stress reaction of cattle. Elevated cortisol concentrations can promote the metabolism of carbohydrates, fat, and protein, provide energy for the body, and ultimately improve the adaptability of cattle to stress [12]. However, excessive cortisol can accelerate protein decomposition and inhibit protein synthesis, resulting in weight loss. It has been reported in beef cattle that cortisol was increased on day 1 but not 6 h after transportation (travel time, 14 h; distance,1000 km; speed, 60–70 km/h) [4], perhaps suggesting that the cortisol level gradually increased after transportation. In this study, there was no significant elevation of cortisol in response to transport treatment, which may be because cortisol was measured 2 h after transportation. Creatine monohydrate was found to be a cortisol blocker; when combined with pyruvate, pyruvyl-creatine is a useful composition comprised of pyruvate and a cortisol blocker [13]. Correspondingly, the present study showed that diet supplemented with RP-CrPyr decreased the serum cortisol concentration of beef cattle, which means that RP-CrPyr could alleviate the transport stress of beef cattle.

During transportation, the imbalance between free radical production and antioxidant mechanisms induces oxidative stress and damage to the body [14]. MDA is the final product of lipid peroxidation and is utilized as an indicator of oxidative stress [15]. T-AOC is composed of antioxidants and antioxidant enzymes and is an important evaluation index that reflects oxidative damage in the body [16]. SOD, GSH-Px, and CAT are major antioxidant enzymes. SOD can scavenge superoxide radicals and convert them into hydrogen peroxide (H_2_O_2_), lipid hydroperoxides are reduced to the corresponding alcohols by GSH-Px, and CAT can decompose H_2_O_2_ into harmless water [17, 18]. As reported previously, transport stress may cause oxidative stress in the brains of chicks by increasing the production of lipid peroxidation and free radicals and reducing the activities of antioxidant enzymes and glutathione [19]. On the other hand, dietary supplementation with creatine reduced the concentration of MDA and increased the activity of SOD in Yili horses [20]. In the current study, transport treatment significantly decreased the serum CAT activity and T-AOC, which means that transportation results in oxidative damage to beef cattle. While dietary supplementation with RP-CrPyr tended to decrease the amount of serum MDA, the result indicates that RP-CrPyr could reduce oxidative stress to some extent. This may be because creatine and pyruvate are both energetic and antioxidant substances [21], and RP-CrPyr can be absorbed in the small intestine in the form of creatine and pyruvate in beef cattle.

TSSBC is reported to be associated with alterations in immune function induced by transport stress and increased exposure to pathogens [22]. In the present study, transport treatment significantly decreased the serum IgA level of beef cattle. It is well known that IgA can eliminate a large number of antigens in a noninflammatory form, which is a very beneficial immune effect for maintaining the internal environment of the body. Moreover, IgA traverses the gut wall from blood into the gut lumen [23]. In mucosal surfaces, IgA binds microbial antigens and influences bacterial gene expression to regulate microbiota growth and epithelial attachment, thereby mediating microbial homeostasis [24]. The reduction in serum IgA levels caused by transport stress in this study might contribute to the decreased immune function and disordered microflora. Correspondingly, group T_CrPyr0 numerically increased rumen Bacteroidota abundance and decreased Firmicutes abundance and significantly increased the *Prevotella* abundance of beef cattle compared with group NT_CrPyr0. Bacteroidota and *Prevotella* are gram-negative bacteria, and LPS is closely bound to the surface of gram-negative bacteria and is released when the bacteria grow rapidly or die [25]. Therefore, the dysbiosis of gram-negative bacteria in the rumen microbiome of beef cattle in group T_CrPyr0 contributed to the elevated levels of rumen and serum LPS. In contrast, in the present study, compared with group T_CrPyr0, group T_CrPyr140 significantly decreased the abundance of rumen Bacteroidota and *Prevotella* and increased Firmicutes abundance, while dietary supplementation with RP-CrPyr decreased the level of serum LPS.

The inflammatory response initiated by LPS could induce the expression of inflammatory cytokines (such as IL-1β, Il-6, IFN-γ, TNF-α) through a series of signal transduction pathways [26, 27]. Previous research found that the contents of TNF-α, IFN-γ, IL-1β, and IL-6 were higher posttransportation than pretransportation [5]. Our data showed that transport treatment decreased the levels of serum IFN-γ, IL-1β, and IL-6 in beef cattle compared with those in nontransport treatment. One reason for this discrepancy in our findings may be that different transport times have different effects on inflammatory cytokines. In addition, it has also been reported that the changes in immune function and cytokines during transportation were not simply rising and falling but a dynamic process [28]. Recently, Zhu et al. [29] identified that extracellular DNA (eDNA) was a nutritional trigger of *Mycoplasma bovis* (*M. bovis*, the main pathogenic microorganism of TSSBC) cytotoxicity, and the release of massive amounts of prokaryotic DNA induced by bacteriophages and antimicrobial drugs was noteworthy. A review showed that increased glucocorticoid release could reduce the diversity and richness of intestinal microorganisms (reviewed by Sherwin et al. [30]); therefore, transport stress may increase the release of eDNA from rumen microbiota and contribute to *M. bovis* proliferation. However, in the present study, there was no significant effect of the transport of RP-CrPyr on the contents of rumen eDNA and serum eDNA.

Rumen fermentation parameters (pH, NH_3_-N, MCP, VFA) are important indicators to evaluate rumen health. Previous studies reported that transportation decreased the rumen fluid pH value [4, 5], but we found that transport treatment tended to increase the pH value. This was because of the interaction of transport and RP-CrPyr on pH. When comparing group T_CrPyr0 individually to group NT_CrPyr0, the pH value was numerically lower in group T_CrPyr0, while group T_CrPyr140 significantly increased the pH value. Rumen NH_3_-N concentration can be influenced by dietary protein breakdown, NH_3_ utilization by microbes, absorption by the rumen wall, and urea hydrolysis in the rumen. Our results showed that transport treatment significantly increased the concentration of NH_3_-N, which may be attributable to the abundance of proteolytic bacteria being increased under transport stress, such as *Prevotella*, which promotes the decomposition of protein in feed and produces ammonia, resulting in the increase of NH_3_-N. Consistently, for dairy cows suffering heat stress, the concentration of rumen NH_3_-N was also increased [31]. VFAs, the main metabolic product of rumen fermentation, provide approximately 70% of the host’s metabolic energy and have a direct relationship with the rumen microbial community [32]. Previous studies reported that transport stress has an influence on ruminal carbohydrate fermentation, but the results have been inconsistent [4, 5]. In the present study, transport treatment significantly decreased the contents of rumen TVFAs, acetate, and butyrate, which may be because beef cattle withdrew feed during transport, thereby reducing the concentration of rumen fermentation substrate and affecting microorganism fermentation and the generation of VFAs. RP-CrPyr supplementation also reduced the amount of TVFA and butyrate compared with RP-CrPyr unsupplemented groups. This may be because RP-CrPyr was decomposed into pyruvate and creatine in the small intestine and absorbed, which can provide energy for the body as energy substances, therefore sparing VFA for energy supply.

One previous study reported that in dairy cattle, preweaning stress did not change the microbial community constantly in the rumen and faeces based on the analysed species richness [33]. Under heat stress, dietary supplementation with CrPyr did not influence the α-diversity index [11]. In this study, to analyse ruminal bacterial richness and diversity indices, we found that there was no difference in the Chao1, Shannon, Simpson, ACE and OTU indices among the four groups. These results were similar to the results of a previous study. In this study, our results indicated that Bacteroidetes, Firmicutes, and Actinobacteriota were the dominant flora at the phylum level among the four groups. Additionally, previous studies reported that Bacteroidetes and Firmicutes were the most dominant flora of ruminal microorganisms in beef cattle [34]. The major classes within the phylum Bacteroidetes in the rumen were found to be hemicellulolytic bacteria, amylolytic bacteria and proteolytic bacteria [35, 36]. The phylum Bacteroidetes has a superb ability to utilize nutrients, including simple and complex sugars and polysaccharides, for its growth to adapt to environmental changes and stresses [37]. Increased phylum Bacteroidetes was also observed in weaning-stressed piglets [38] and heat-stressed birds [39, 40]. The phylum Firmicutes contains a large number of cellulolytic bacteria and thus has a stronger fibre decomposition ability [41, 42]. During heat stress, ruminal cellulolytic bacteria were downregulated, while amylolytic bacteria were upregulated in dairy cows [43]. Similarly, in the present study, transport stress numerically increased Bacteroidetes phylum (containing amylolytic bacteria) abundance and decreased Firmicutes (containing cellulolytic bacteria) abundance. Furthermore, the *Prevotella* genus was the predominant genus belonging to the Bacteroidetes phylum, and its level was significantly increased in beef cattle within T_CrPyr0. In contrast, transported beef cattle fed RP-CrPyr had lower levels of the Bacteroidetes phylum and *Prevotella* genus and higher levels of the Firmicutes phylum, which means that RP-CrPy promotes the restoration of normal rumen flora under transport stress. Recently, there has been increasing concern about the relationship between stress and gut microbiota. Depression patients had an increased plasma cortisol level [44], a higher proportion of Bacteroidetes and a lower proportion of Firmicutes [45] and upregulation of *Prevotella* genus abundance in the gut microbiome [46]. Therefore, transport stress caused an increase in the abundance of Bacteroidetes and *Prevotella* in the rumen microbial community, which may be related to increased serum cortisol levels, while RP-CrPy reduced the cortisol level of transported beef cattle, which can stimulate the restoration of the natural flora.

## Conclusion

In conclusion, transport stress elicited negative changes in endocrine parameters, antioxidant status, immunity, rumen fermentation and microorganisms of beef cattle. Dietary supplementation with RP-CrPyr might be beneficial to alleviate transport stress by decreasing serum cortisol and LPS levels and promoting the restoration of the rumen natural flora.

## Methods

### Animal treatments and experimental Diets

Twenty male Chinese Simmental crossbred cattle (initial body weight = 659 ± 16 kg) of 18 months of age were randomly allocated to four groups using a 2 × 2 factorial arrangement with two RP-CrPyr supplemental levels (0 or 140 g/d) in basal diets and two types of transport (5 min or 12 h). Compared to the 12 h of transportation, the 5 min of transportation was relatively short; for ease of presentation and understanding, 5 min of transportation was described as nontransport. That were T_CrPyr140 (12 h transport + 140 g/d RP-CrPyr in basal diet); T_CrPyr0 (12 h transport + 0 g/d RP-CrPyr in basal diet); NT_CrPyr140 (5 min transport + 140 g/d RP-CrPyr in basal diet); NT_CrPyr0 (5 min transport + 0 g/d RP-CrPyr in basal diet). After feeding for 30 days, three animals from each treatment were selected for euthanasia and to collect samples. The cattle in the nontransport group were fed at 16:00 pm on September 16, 2020, loaded onto trucks (at 04:00 am on September 17, 2020) to slaughter near the farm (1 km), which took approximately 5 min. After unloading, the cattle rested for 2 h and were slaughter by electrical stunning and exsanguination (at 06:00 am on September 17, 2020). Briefly, the cattle in the nontransport group were fasted for approximately 14 h (12 h fasting prior transport, 5 min transport, 2 h lairage followed by slaughter). The transported beef cattle were fed at 06:00 am on September 16, 2020 and transported from Xuchang, Henan Province, to Nanyang, Henan Province, for 12 h at an average speed of 60–70 km/h. The journey started at 13:00 pm on September 16, 2020, and the cattle arrived at 01:00 am on September 17, 2020. The outside temperature was between 22 and 32 °C with a relative humidity of 30–72%. After unloading, the cattle were allowed to rest for 2 h and then electrical stunning and exsanguination (03:00 am on September 17, 2020). Briefly, the cattle in the transport group were fasted for approximately 21 h (7 h fasting prior transport, 12 h transport, 2 h lairage followed by slaughter).

CrPyr (purity of 99.9%) was purchased from Shanghai Jinli Technology Co., Ltd. (Shanghai, China). The production of RP-CrPyr was entrusted to Hangzhou King Techina Feed Co., Ltd. (Hangzhou, China), the CrPyr coating rate was 40%, the rumen bypass ratio (12 h) was 82.8%, and the dissolution of intestinal fluid (12 h) was 78.6%. Our previous study results indicated that Jinjiang cattle fed 60 g/d CrPyr had a better resistance to heat stress [11]. Therefore, the present experiment selected a 140 g/d dose to explore the regulatory mechanism of RP-CrPyr in beef cattle. RP-CrPyr was added to the concentrates, divided into two daily feeds (06:00 and 16:00). After the rice straw was fed, concentrate was given. All cattle were housed in individual solid concrete floor pens in a closed cowshed, while clean, freshwater was available at all times. The composition and nutrient levels of the experimental diet are shown in Table 5.

**Table 5:** Composition and nutrient levels of experimental diet (air-dry basis, %)

Ingredients	Content	Nutrient levels	Content
Wheat Straw	40.00	Dry matter	87.20
Corn	43.74	Crude protein	13.52
wheat bran	3.66	Crude fat	2.47
Soybean meal	9.12	Ash	7.86
Sodium bicarbonate	0.42	Neutral detergent fiber	36.18
Premix^a^	3.06	Acid detergent fiber	15.81
Tatol	100		

^a^The premix (per kg of diet) is: 80000 IU of vitamin A, 20000 IU of vitamin D3, 280 mg of vitamin E, 4100 mg of Fe, 1100 mg of Mn, 800 mg of Zn, 265 mg of Cu, 120 g of Ca and 35 g of P

### Sample collection

Before slaughter, 15 mL of blood sample was taken from the jugular vein of cattle into evacuated nonanticoagulative tubes. The blood samples were centrifuged at 3000 g (10 min, 4 °C) to collect serum samples, and then stored at − 20 °C to measure indices. Then, the beef cattle were slaughtered by electrical stunning and exsanguination. After slaughter, 400 mL of rumen fluid was collected from the upper, middle, and lower sites in the rumen and squeezed through four layers of gauze. The rumen fluid was immediately measured for pH using a portable pH metre (HANNA Instruments, Cluj-Napoca, Romania). Then, the samples were divided into three portions. The first 8 mL of rumen fluid was mixed with 2 mL of 25% (wt/vol) metaphosphoric acid and used for VFA analysis. One subsample (10 mL) of rumen fluid was mixed with 2 mL of H_2_SO_4_ (1% vol/vol) for determination of ammonia nitrogen (NH_3_-N), and another subsample (10 mL) of rumen fluid was used for microbial crude protein (MCP) and 16S rDNA sequencing analysis. These samples were frozen in liquid nitrogen and stored at − 80 °C until DNA extraction.

### Chemical analyses

The VFA concentrations in the rumen fluid samples were determined using gas chromatography (Shimadzu GC-2014, Japan) equipped with a capillary column (Stabilwax, Restek, Bellefonte, PA, USA). The NH_3_-N concentration was measured using a TU-1901 spectrophotometer (Beijing Purkinje General Instrument Co. Ltd., China) according to a method described by Broderick and Kang [47]. MCP production was determined according to the method of Coomassie Brilliant Blue [48].

Serum cortisol, triiodothyronine (T3), thyroxine (T4), IgG, IgA, IgM, tumour necrosis factor-α (TNF-α), interferon γ (IFN-γ), interleukin-1 (IL-1β), IL-6, lipopolysaccharide (LPS) and rumen fluid LPS were determined using commercial kits (Nanjing Jiancheng Bioengineering Institute, Nanjing, China). The level of malondialdehyde (MDA), the activities of superoxide dismutase (SOD), glutathione peroxidase (GSH-Px), catalase (CAT) and total antioxidant capacity (T-AOC) in the serum samples were measured using commercial kits (Nanjing Jiancheng Bioengineering Institute). Serum and rumen fluid eDNA content analysis was assessed by a dsDNA HS Assay Kit for Qubit® (Kit: Shanghai Yisheng Bioengineering Institute, Shanghai, China).

### 16S rDNA sequencing analysis

According to manufacturer, microbial community genomic DNA was extracted from ruminal fluid samples using the E.Z.N.A.® soil DNA Kit (Omega Bio-tek, Norcross, GA, U.S.). The DNA extract was checked on 1% agarose gel, and DNA concentration and purity were determined with NanoDrop 2000 UV-vis spectrophotometer (Thermo Scientific, Wilmington, USA). The hypervariable region V3-V4 of the bacterial 16S rRNA gene was amplified with primer pairs 338F (5′-ACTCCTACGGGAGGCAGCAG-3′) and 806R (5′-GGACTACHVGGGTWTCTAAT-3′) by an ABI GeneAmp® 9700 PCR thermocycler (ABI, CA, USA). The PCR amplification of 16S rRNA gene was performed as follows: initial denaturation at 95 °C for 3 min, followed by 27 cycles of denaturing at 95 °C for 30 s, annealing at 55 °C for 30 s and extension at 72 °C for 45 s, and single extension at 72 °C for 10 min, and end at 4 °C. The PCR mixtures contain 5 × *TransStart* FastPfu buffer 4 μL, 2.5 mM dNTPs 2 μL, forward primer (5 μM) 0.8 μL, reverse primer (5 μM) 0.8 μL, *TransStart* FastPfu DNA Polymerase 0.4 μL, template DNA 10 ng, and finally ddH_2_O up to 20 μL. PCR reactions were performed in triplicate. The PCR product was extracted from 2% agarose gel and purified using the AxyPrep DNA Gel Extraction Kit (Axygen Biosciences, Union City, CA, USA) according to manufacturer’s instructions and quantified using Quantus™ Fluorometer (Promega, USA).

Purified amplicons were pooled in equimolar and paired-end sequenced (2 × 300) on an Illumina MiSeq platform (Illumina, San Diego, USA) according to the standard protocols by Majorbio Bio-Pharm Technology Co. Ltd. (Shanghai, China). The raw reads were deposited into the NCBI BioProject database (Accession Number: PRJNA732599).

The raw 16S rDNA gene sequencing reads were demultiplexed, quality-filtered by Trimmomatic and merged by FLASH with the following criteria: (i) the 300 bp reads were truncated at any site receiving an average quality score of < 20 over a 50 bp sliding window, and the truncated reads shorter than 50 bp were discarded, reads containing ambiguous characters were also discarded; (ii) only overlapping sequences longer than 10 bp were assembled according to their overlapped sequence. The maximum mismatch ratio of overlap region is 0.2. Reads that could not be assembled were discarded; (iii) Samples were distinguished according to the barcode and primers, and the sequence direction was adjusted, exact barcode matching, 2 nucleotide mismatch in primer matching.

Operational taxonomic units (OTUs) with 97% similarity cutoff were clustered using UPARSE (version 7.1, http://drive5.com/uparse/), and chimeric sequences were identified and removed. The taxonomy of each OTU representative sequence was analyzed by RDP Classifier (http://rdp.cme.msu.edu/) against the 16S rRNA database (eg. Silva 132/16s_bacteria) using confidence threshold of 0.7.

### Statistical analysis

The data were analysed with SPSS (version 17.0, IBM, Armonk, NY, USA). The four treatment groups were analysed as a 2 × 2 factorial arrangement using a general linear model (GLM) to elucidate the main effects of transport, RP-CyPry, and their interactions. The results are shown as the mean and standard error mean (SEM). When the interaction was significant, differences among means were determined using Tukey’s multiple range test for individual comparisons. A *P* value < 0.05 was considered statistically significant.

## Data Availability

The datasets analysed during the current study are available from the corresponding author on reasonable request.

## Abbreviations

CAT: Catalase
eDNA: Extracellular DNA
GSH-Px: Glutathione peroxidase
HPA: Hypothalamic-pituitary-adrenal
IFN-γ: Interferon γ
IL-1β: Interleukin-1β
IL-6: Interleukin-6
LPS: Lipopolysaccharide
MCP: Microbial crude protein
MDA: Malondialdehyde
NH_3_-N: Ammonia nitrogen
OTUs: Operational taxonomic units
RP-CrPyr: Rumen-protected creatine pyruvate
SEM: Standard error of the mean
SNS: Sympathetic nervous system
T3: Triiodothyronine
T4: Thyroxine
TSSBC: Transport stress syndrome of beef cattle
SOD: Superoxide dismutase
T-AOC: Total antioxidant capacity
TNF-α: Tumour necrosis factor-α
TVFA: Total volatile fatty acids
